# Glycopolypeptoids as Novel Biomimetic Antifreeze Agents: Structural Design, Synthesis, and Antifreeze Properties

**DOI:** 10.3390/polym17121600

**Published:** 2025-06-08

**Authors:** Liugen Xu, Junwei Pi, Lei Feng, Junhao Wen, Minghai Zhao, Amjad Ali, Jianwei Lu, Li Guo

**Affiliations:** 1 School of Materials Sciences and Engineering, Jiangsu University, Zhenjiang 212013, China; 2 College of Materials Science and Engineering, Nanjing Forestry University, Nanjing 210037, China

**Keywords:** glycopolypeptoids, cryopreservation, antifreeze properties

## Abstract

Glycopolypeptoids were synthesized and showed effective antifreeze activity, demonstrating their potential as novel antifreeze agents for cryopreservation. These polypeptide analogs offer improved stability and tunability compared with natural antifreeze glycoproteins (AFGPs) and existing synthetic mimics. Using the ring-opening polymerization of N-substituted N-carboxyanhydride monomers followed by click chemistry, glycopolypeptoids with controlled polymerization degrees and varied structures were designed and prepared. Their antifreeze performance was assessed via nanoliter osmometry and ice recrystallization inhibition assays, revealing a strong correlation between the molecular structure and antifreeze activity. The findings highlight glycopolypeptoids as a promising, cost-effective alternative to natural AFGPs, providing new insights into the development of biomimetic cryoprotectants. This study expands the understanding of synthetic antifreeze materials and offers a practical approach for improving cryopreservation efficiency in biomedical and industrial applications.

## 1. Introduction

Cryopreservation is widely used in food storage, the pharmaceutical industry, and scientific research [1,2]. Ice is one of the major challenges in cryopreservation. Ice formation, growth, and recrystallization during the process can cause severe damage, resulting in a loss of cell function [3]. Many plants, fish, and insects, however, can survive below 0 °C in nature due to the existence of antifreeze glycoproteins (AFGPs).

AFGPs can protect living organisms from the damage of ice [4,5,6,7], including by tuning ice nucleation [8], inhibiting ice growth and recrystallization [9,10], reducing the freezing point [11], adhering to the ice [12], and retaining a liquid environment [13]. AFGPs could be ideal antifreeze agents in cryopreservation. However, it is extremely difficult and expensive to separate AFGPs from organisms, which limits the large-scale production and application of AFGPs. Therefore, antifreeze agents with simple preparation, a low cost, and good antifreeze performance are urgently needed to replace natural AFGPs.

Synthetic peptides mimicking AFGPs as antifreeze agents have been reported. Ben et al. synthetized C-linked analogs of native AFGPs (C-AFGPs) and evaluated their antifreeze performance [14,15]. Sumii et al. [16] synthetized galactose–proline oligomers and studied their physical properties. Qin et al. [17] synthesized L-proline oligomers (L-Pron) with the same structure as AFGPs and tested their effects on oocytes via cryopreservation. Deleray et al. [18] presented a route to synthetic AFGPs (sAFGPs) using N-carboxyanhydride polymerization and tested their antifreeze performance. Although peptide antifreeze agents prepared by mimicking AFGPs have some antifreeze activity, they have not been widely used due to the disadvantages that they have, including difficulties in synthesis and being readily cleaved by enzymes.

Polypeptoids are novel polypeptide analogs that can overcome many of the disadvantages associated with polypeptides, such as instability with proteases [19]. In contrast to polypeptides, polypeptoids lack the chiral center in the skeleton and do not have extensive hydrogen bond interactions due to nitrogen substitution. Therefore, the conformation of polypeptoids is primarily determined by the spatial and electronic properties of side chains [20,21,22,23]. It has been proven that polypeptoids exhibit biodegradability, biocompatibility, and thermal processability, making them promising for various biotechnological applications [24,25,26]. It has been reported that polypeptoids can affect crystal formation and growth [27,28]. Furthermore, our earlier studies indicated that polypeptoids have the ability to tune ice crystal morphology and inhibit ice recrystallization, which suggests the great potential of polypeptoids as antifreeze material [29,30,31]. However, an exploration of the antifreeze properties of glycopolypeptoids mimicking the structures of AFGPs has not been reported.

Herein, glycopolypeptoids were synthesized and their antifreeze properties were studied for the first time. A series of glycopolypeptoids with different structures were designed and prepared by the ring-opening polymerization (ROP) of N-substituted N-carboxyanhydride (NNCA) monomers followed by click chemistry. The degrees of polymerization could be controlled by the initiator-to-monomer feeding ratio. Different copolymers were obtained by varying the monomer feeding sequence. Click chemistry was employed to introduce different sugars to polymer side chains. The antifreeze properties of the prepared glycopolypeptoids were evaluated by nanoliter osmometer and ice recrystallization experiments. The structure–property relationships were also studied. The goals of this study were to explore the potential of glycopolypeptoids as antifreeze protectants in cryopreservation and provide experimental guidance for the structural design of novel biomimetic antifreeze agents.

## 2. Materials and Methods

### 2.1. Materials

2-Azidoethyl 2, 3, 4, 6-Tetra-O-acetyl-β-D-glucopyranoside, 1, 3, 4, 6-Tetra-O-acetyl- 2-azido-2-deoxy-β-D-galactopyranose, and 2-Acetamido-3, 4, 6-tri-O-acetyl-2-deoxy-β-D-glucopyranosyl were provided by Macklin. Anhydrous magnesium sulfate, acetone, ethyl acetate, anhydrous ethylamine, ether, hydrochloric acid (38%), methanol, and ethanol were purchased from the Sinopharm Chinese Reagent Company. Propargylamine, triethylamine, glyoxylic acid, glyoxylic acid monohydrate, phosphorus trichloride, di-tert-butyl dicarbonate, dimethylformamide (DMF), sodium methoxide, isobutylamine, pentamethyldiethylenetriamine (PMDETA), cuprous bromide, sodium chloride, and ethylene diamine tetraacetic acid were supplied by Aladdin. Dichloromethane (DCM), n-hexane, and tetrahydrofuran (THF) were purchased from Macklin and purified by a purification system before use as required. Mouse fibroblasts (L929) were purchased from MeiSen Cell Biotechnology LTD. Deuterated chloroform and trifluoroacetic acid as well as the culture medium, trypsin, fetal bovine serum, and Cell Counting Kit-8 (CCK-8) were bought from Adamas.

### 2.2. Synthesis of Polypeptoids

#### 2.2.1. Synthesis of Ethyl-NCA (M_1_)

Glyoxylic acid monohydrate (22.5 g; 240 mmol) and anhydrous ethylamine (5.4 mL; 120 mmol) were added to CH_2_Cl_2_ (600 mL) and thoroughly stirred at room temperature overnight. The reaction mixture was dried with a rotovap, and 1 M of a HCl aqueous solution (480 mL) was added. The reaction solution was stirred at 120 °C under reflux conditions overnight. The volatile part of the resulting mixture was evaporated and the residue was recrystallized in methanol/ethyl ether to produce a white solid (9.7 g; 60% yield).

A round-bottomed flask was filled with the above product (9.7 g; 69.5 mmol), di-tert-butyl dicarbonate (23 g; 105 mmol), triethyl amine (48.3 mL; 347.5 mmol), and distilled water (270 mL). The mixture was stirred at room temperature overnight and extracted with hexane (2 × 270 mL). The aqueous phase was poured into 4 M of a HCl aqueous solution (270 mL) to ensure the pH of the solution was less than 2, and then extracted with ethyl acetate (3 × 80 mL). The organic phase was washed with brine (270 mL), separated, and dried over anhydrous MgSO_4_. After filtration, the solvent was evaporated to produce a yellow oil (11.6 g; 83% yield).

The above product (11.6 g; 57 mmol) was dissolved in anhydrous CH_2_Cl_2_ (270 mL) under a nitrogen atmosphere, and PCl_3_ (6 mL; 69 mmol) was added dropwise to the solution at 0 °C. The reaction mixture was stirred for 2 h and the solvent was removed under vacuum. The residue was transferred to a glove box, and a yellow oily product, Ethyl-NCA (**M_1_**), was obtained through purification in anhydrous CH_2_Cl_2_/hexane and THF/hexane (5.52 g; 75% yield). The ^1^H NMR spectrum of **M_1_** is presented in Figure S1.

#### 2.2.2. Synthesis of Propargyl-NCA (M_2_)

Glyoxylic acid (32.2 g; 350 mmol) and propargyl amine (9.0 mL; 140 mmol) were added to CH_2_Cl_2_ (350 mL) and thoroughly mixed at room temperature overnight. The reaction mixture was dried with a rotovap, and 1 M of a HCl aqueous solution (224 mL) was added. The reaction solution was heated under reflux overnight. The volatile part of the resulting mixture was evaporated and the residue was recrystallized in methanol/ethyl ether to produce a brown solid (15.1 g; 72% yield).

A round-bottomed flask was filled with the above product (15.1 g; 100.1 mmol), di-tert-butyl dicarbonate (33 g; 150.2 mmol), triethyl amine (70 mL; 500.5 mmol), and distilled water (336 mL). The mixture was stirred at room temperature overnight and extracted with hexane (2 × 390 mL). The aqueous phase was poured into 4 M of a HCl aqueous solution (98 mL) to ensure the pH of the solution was less than 2, and then extracted with ethyl acetate (3 × 98 mL). The organic phase was washed with brine (390 mL), separated, and dried over anhydrous MgSO_4_. After filtration, the solvent was evaporated to produce a brown solid (16.4 g; 77% yield).

The above product (16.4 g; 77 mmol) was dissolved in anhydrous CH_2_Cl_2_ (256 mL) under a nitrogen atmosphere, and PCl_3_ (8.05 mL; 92 mmol) was added dropwise to the solution at 0 °C. The reaction mixture was stirred for 2 h and the solvent was removed under vacuum. The residue was transferred to a glove box, and a product, Propargyl-NCA (**M_2_**), was obtained through purification in anhydrous CH_2_Cl_2_/hexane and THF/hexane (6.44 g; 65% yield). The ^1^H NMR spectrum of **M_2_** is presented in Figure S2.

#### 2.2.3. Synthetic Procedure for the Poly[(*N*-ethyl glycine)-r-(*N*-propargyl glycine)] Random Copolymer PNEG-r-PNPG

In a glovebox, the calculated volume of a ^i^BuNH_2_/THF stock solution (0.2 M; 204 µL) was added to a THF solution of Py-NCA (140 mg, 1 mmol, and 0.8 M) and Et-NCA (260 mg, 2 mmol, and 0.6 M). The reaction was stirred at 50 °C and monitored by FT-IR until all monomers were converted (Figure S3). The final random copolypeptoid was precipitated out in excess hexane and then isolated and dried (243 mg; 88% yield).

#### 2.2.4. Synthetic Procedure for the Poly[(*N*-ethyl glycine)-b-(*N*-propargyl glycine)] Block Copolymer PNEG-b-PNPG

In a glovebox, Et-NCA (212 mg, 1.64 mmol, and 0.6 M) was dissolved in anhydrous THF, followed by the addition of a stock solution of ^i^BuNH_2_ (333.2 µL; 0.2 M). The reaction was stirred at 50 °C and monitored by FT-IR until the monomer was fully converted. Then, Py-NCA (223 mg; 1.6 mmol) was added to the reaction solution and stirring was continued at 50 °C until all the Py-NCA was consumed. The final block copolypeptoid was precipitated out in excess hexane and then isolated and dried (255 mg; 85% yield).

#### 2.2.5. Synthetic Procedure for the Glycopolypeptoids

In a glovebox, a measured amount of Azido sugar (287 mg, 0.69 mmol, and [N_3_]_0_/[propargyl]_0_ = 1.2), PNEG_51_-r-PNPG_26_ (62.1 mg; 9 µmol), and CuBr/PMDETA (248 mM, 311 µL, and [Cu]_0_/[PMDETA]_0_/[propargyl]_0_ = 33:33:100) were dissolved in 2 mL DMF. After stirring at 50 °C for 24 h, the mixture was dialyzed in pure water for 24 h (dialysis tube: MD44-2000). The reaction mixture was then lyophilized to yield a light grayish-green solid. The product (265 mg; 17.7 µmol) and sodium methoxide (8 mg, 0.15 mmol, and [-OAc]_0_/[CH_3_ONa]_0_ = 12) were added to ultra-dry methanol and stirred into an ice–water mixture for 3.5 h. The reaction mixture was dialyzed and lyophilized to yield a light grayish-green solid (105 mg; 60% yield).

### 2.3. Characterization of Polypeptoids and Glycopolypeptoids

The polymer structures were confirmed using ^1^H NMR (Bruker, 400 MHz; Billerica, MA, USA) spectroscopy. The peaks were referred to in parts per million (ppm) relative to the proton impurities of Chloroform-d (CDCl_3_) or Deuterium oxide (D_2_O). The chemical bond compositions of monomers and polymers were characterized by employing an attenuated total reflection–Fourier transform infrared spectrometer (Thermo Fisher, ATR-FTIR, Nicolet 8700, Waltham, MA, USA). Tandem gel permeation chromatography (GPC) adopted the 1525 μ (Worcester County, MA, USA) gel permeation chromatograph of the Waters Corporation of the United States, equipped with a 2414 differential refractive light detector. LiBr/DMF (0.05 M) was used as a flowing solution at a flow rate of 1 mL/min. The column temperature was 50 °C and the detector sample cell was 40 °C. The calibration curve for the molecular weight analysis was prepared using polystyrene as the standard.

### 2.4. Characterization of Antifreeze Activity

#### 2.4.1. Nanoliter Osmometry Experiment

The preferred method of testing the ice crystal morphology, growth rate, and thermal hysteresis (TH) of a single ice crystal with different degrees of supercooling temperature is nanoliter osmometry, which can accurately control water droplets, relative humidity, and temperature with accuracy of 0.01 °C [32,33]. Briefly, the solution containing glycopolypeptoids was injected into a six-hole sample holder with silicone oil on a thermal console fixed onto the optical table of a microscope (Nikon, LV100ND, Tokyo, Japan). The experiment employed a nanoliter osmometer (OsmoONE, INSTEC, Boulder, CO, USA) and a high-speed camera (PCO dimax S1, Excelitas PCO, Gottingen, Germany). The glycopolypeptoid-solution droplet dispersed in the oil and was quickly frozen to −18 °C to form an ice slurry; this was then slowly heated to the melting temperature until a single ice crystal (approximately 15 microns in diameter) was left, where the temperature was defined as the melting temperature (T_m_). Then, the thermal control station was slowly cooled to the required supercooling temperature (ΔT) and the ice crystal growth process was recorded by a high-speed camera to calculate the ice crystal growth rate and observe the ice morphology. The experiment was repeated at least 3 times for each ΔT of each sample. If the measured sample had a TH gap, the size of the ice crystal remained unchanged and the temperature was lowered to the temperature at which a single ice crystal could grow significantly. This temperature was defined as the freezing temperature (T_f_). The TH value was the difference between T_m_ and T_f_.

#### 2.4.2. Ice Recrystallization Inhibition (IRI) Experiment

Ice recrystallization inhibition (IRI) was used to detect the capability of AFGPs to restrain ice crystal growth without influencing ice nucleation or the freezing temperature. Two adjacent ice crystals can recrystallize and grow larger ice crystals at the expense of smaller ice crystals to minimize the total surface energy through Ostwald ripening or grain boundary migration mechanisms [34,35]. In the case of recrystallization inhibitors that slow down or prevent the ripening process, the most common and simple technique is to quantitatively assess IRI activity using a “splat” assay, as shown by Knight [36,37,38]. The device is equipped with a Linkam freezing table (C194, INSTEC, Boulder, CO, USA) and a Nikon polarized optical microscope (Nikon, LV100ND, Tokyo, Japan), which can be sealed to obtain a certain humidity. Droplets of 10 µL of the glycopolypeptoid solution dissolved in a PBS buffer at concentrations of 0.2 mg mL^−1^, 0.5 mg mL^−1^, 1.0 mg mL^−1^, and 2.0 mg mL^−1^ were dropped from a height of 1.5 m onto a stage pre-cooled to −60 °C, forming a thin solid ice film. Subsequently, the stage was warmed up to −8 °C at a rate of 5 °C min^−1^ and kept at −8 °C for 30 min to allow ice recrystallization and observe IRI activity. A high-speed camera (PCO dimax S1, Excelitas PCO, Gottingen, Germany) was used to record photomicrographs of the samples to obtain the two largest orthogonal dimensions across the surface of the ice crystal grains. In the observation field, Nano Measurer was used to measure the crystal grain size of all ice crystals, among which the 10 largest crystal grain sizes were selected and the average value was taken to quantitatively evaluate the recrystallization activity of the ice. For every sample, the corresponding experimental steps were repeated at least three times.

### 2.5. Cytotoxicity Test

The cytotoxicity of the glycopolypeptoids was determined using a Cell Counting Kit-8 (CCK-8) or MTT assay, as previously reported [39,40,41]. Briefly, NCTC clone 929 (L-929) inoculated into 96-well plates was cultivated with 100 µL of a complete medium containing glycopolypeptoids at a concentration of 1 mg mL^−1^ in a growth medium with Van-M (18 mM). After cells were incubated for 24 h at 37 °C and 5% CO_2_, 10 µL of the CCK-8 solution was added and incubated for 1–4 h before measuring absorbance at 450 nm using a microplate spectrophotometer (Synergy H1, Bio-Tek, Winooski, VT, USA). Each sample was tested in six parallel replicates. The relative viability of the L929 cells (σ) was calculated by the following formula (Equation (1)):

(1) $$\sigma \;=\;\frac{\beta_{1}-\beta_{0}}{\beta_{2}-\beta_{0}}\;\times \;100\%$$

where $\beta_{0}$ stands for the average OD_450_ value of solutions containing only the culture solution and no cells; $\beta_{1}$ stands for the average OD_450_ value of solutions containing a glycopolypeptoid sample, cells, and the culture solution; and $\beta_{2}$ stands for the average OD_450_ value of the solutions containing cells and the culture solution but without glycopolypeptoids.

## 3. Results and Discussion

### 3.1. Results of the Characterization of Polypeptoids and Glycopolypeptoids

To study the structure–property relationship, a series of block and random copolypeptoids with different molecular weights, i.e., different degrees of polymerization (DPs), were designed by mimicking the structures of AFGPs. It is believed that both hydrophobic and hydrophilic moieties in AFGPs are necessary for antifreeze functions. Herein, ethyl groups were introduced to polypeptoid side chains as the hydrophobic units. Alkyne groups were introduced for post-polymerization to connect sugars onto the polypeptoid side chains as the hydrophilic units. Block and random copolypeptoids could be obtained by sequential monomer feeding and mixed monomer feeding, respectively. The polymer molecular weight could be controlled by tuning the monomer-to-initiator feeding ratios.

**Scheme 1:**
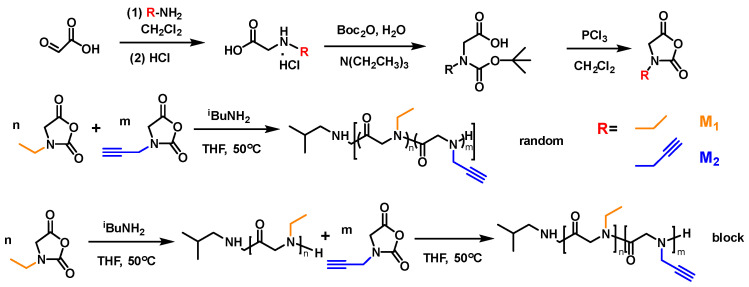
The synthetic routes towards M_1_ and M_2_ monomers and polypeptoids.

**Scheme 2:**
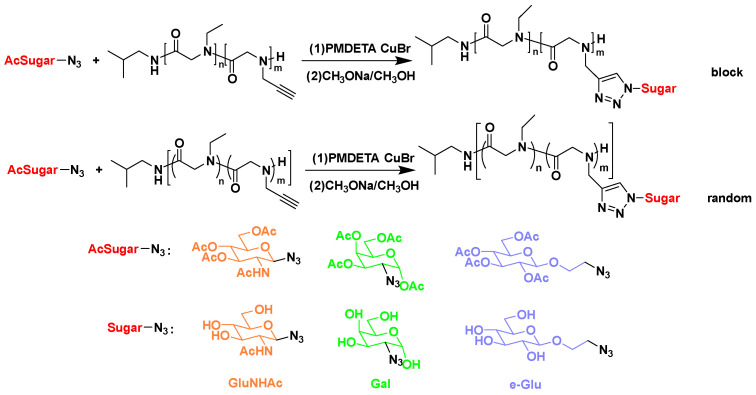
The synthetic routes towards block and random glycopolypeptoids.

**Figure 1:**
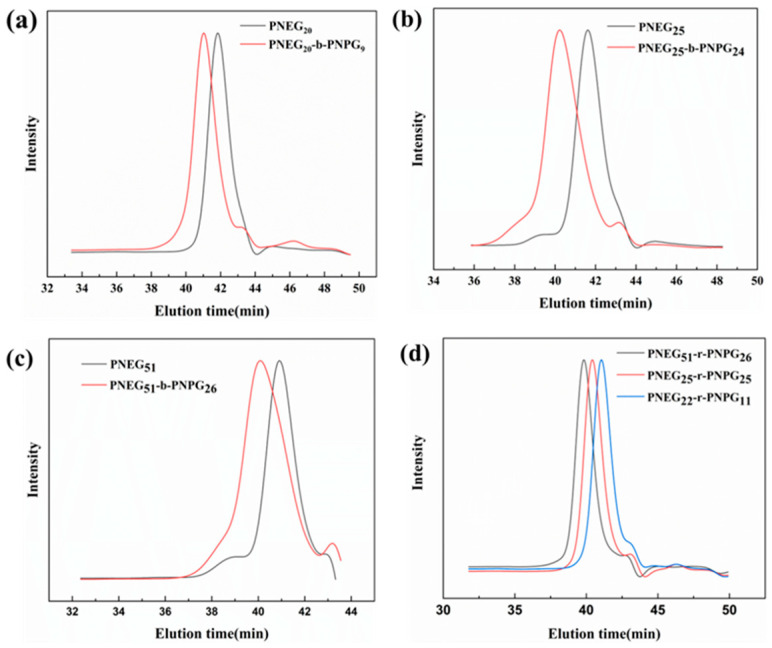
GPC characterization: (**a**–**c**) comparison of GPC traces of one-block polypeptoids and two-block polypeptoids; (**d**) GPC traces of PNEG-r-PNPG random copolypeptoids with different DPs.

**Figure 2:**
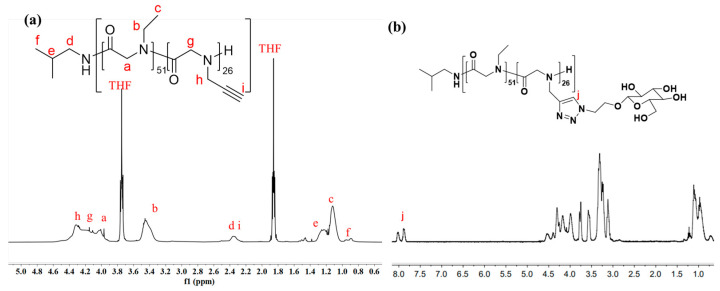
^1^H NMR spectra of (**a**) PNEG_51_-r-PNPG_26_ (400 MHz; CDCl_3_) and (**b**) corresponding glycopolypeptoids (PE_51_-r-(PG-e-Glu)_26_ (400 MHz; D_2_O)).

First, monomers M_1_, with the ethyl group, and M_2_, with alkyne group, were synthesized using the reported method and characterized by ^1^H NMR to confirm the structures [42]. With ^i^BuNH_2_ as the initiator, block copolypeptoids were synthesized by sequentially adding M_1_ and M_2_ and random copolypeptoids were synthesized by simultaneously adding M_1_ and M_2_ (Scheme 1). The polymerizations were monitored by FT-IR. The disappearance of monomer peaks at 1850 cm^−1^ and 1760 cm^−1^, as well as the formation of a polymer peak at 1670 cm^−1^, indicated the full conversion of the monomers into a polymer (Figure S2). The polymers were characterized by GPC, and monomodal distribution was observed (Figure 1 and Table 1) [3]. Polymers with higher DPs, i.e., a higher molecular weight, eluted earlier than the lower ones. For the block copolypeptoids, the full shift of the PNEG peak to the PNEG-b-PNPG peak indicated that all PNEG chains had extended to form the block copolymer PNEG-b-PNPG (Figures S4 and S5). The prepared polymers were characterized by ^1^H NMR to confirm the structures (Figure 2a). The DPs were obtained by calculating the proton integration ratio of the side chain to the end group from the following formula (Equation (2)):

(2) $${DP}_{PNEG}=\frac{(S2/2)}{(S1/6)}$$

$${DP}_{PNPG}=\frac{(6\times S3/S1)-2}{1}$$

where S1 stands for the integration of peak f, S2 stands for the integration of peak b, and S3 stands for the integration of peaks d and i. All the prepared copolypeptoids are summarized in Table 1. The experimental DPs from ^1^H NMR agreed very well with the theoretical values from the feeding ratios, suggesting good control of the polymerization. The M_n_ from GPC was quite different from that from ^1^H NMR, which was due to the intrinsic differences in the two characterization methods. From ^1^H NMR, the DP could be calculated from the integrations of the end groups and repeating units. The M_n_ was then calculated and obtained from the DP. Therefore, the M_n_ from ^1^H NMR was the absolute molecular weight. The M_n_ obtained from GPC, however, was a relative molecular weight calibrated against polystyrene standards (Figure S6). Therefore, it reflected the molecular weight of the sample as if it had the same hydrodynamic volume as polystyrene of a given size. It did not represent the absolute molecular weight of the polymer. After polymerization, click chemistry was employed to connect sugars to polypeptoid side chains by the reaction of azide and alkyne (Scheme 2). As shown in Figure 2b and Figure S7, the appearance of triazole ring peaks suggested the successful conjugation of sugars and glycopolypeptoids.

### 3.2. Effect of Glycopolypeptoids on Ice Crystal Morphology

**Figure 3:**
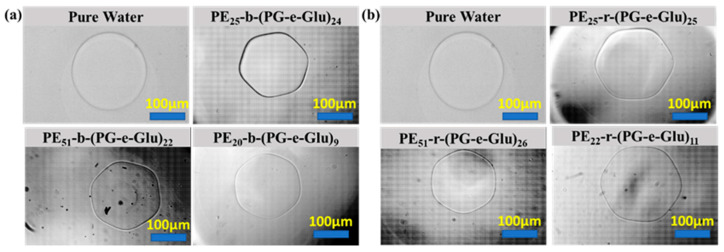
Optical images of ice crystal morphology (ΔT = 0.06 °C; pure water is the blank control): (**a**) the block glycopolypeptoid solutions; (**b**) the random glycopolypeptoid solutions.

The prepared glycopolypeptoids were dissolved in pure water at varying concentrations to conduct the nanoliter osmometer experiment. This test allowed for the observation of the ice crystal morphology and the growth process of a single ice crystal under different subcooling temperatures (ΔT). Thermal hysteresis (TH) is the difference between the equilibrium melting and freezing temperatures of ice crystals, which is not desirable for antifreeze agents used in cryopreservation [43,44]. It was noticed that TH was not observed for all glycopolypeptoid samples, which suggested that glycopolypeptoids could be an effective antifreeze agent in cryopreservation. In pure water, the ice crystal exhibited a flat disc shape. However, the presence of glycopolypeptoids consistently altered the morphology to a hexagonal shape, regardless of the main-chain sequence, molecular weight, or the ratio of hydrophobic-to-hydrophilic units (Figure 3).

**Figure 4:**
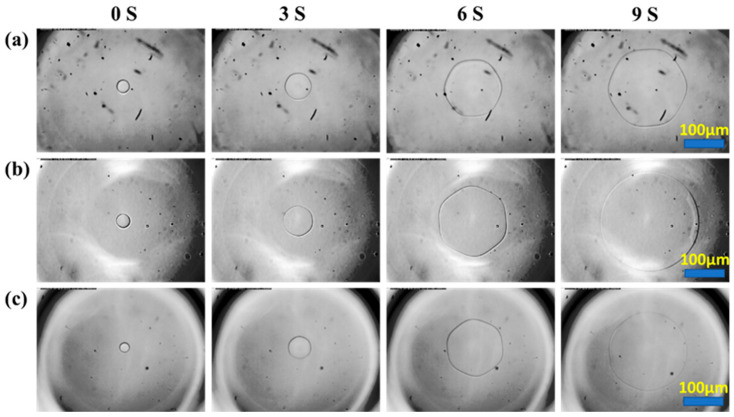
Dynamic ice-shaping process of different glycopolypeptoid solutions (ΔT = 0.1 °C; 2 mg mL^−1^): (**a**) PE_25_-r-(PG-GluNHAc)_25_; (**b**) PE_25_-r-(PG-Gal)_25_; (**c**) PE_25_-r-(PG-e-Glu)_25_.

To further study the tuning process of glycopolypeptoids to ice crystals, the growing process of single ice crystals in glycopolypeptoid solutions was recorded by a high-speed camera in a nanoliter osmometer experiment (Figure 4). At the beginning, it was hard to ascertain if the ice crystals had a disc shape or a hexagonal shape because the crystals were too small. Rapidly, the ice crystals in all glycopolypeptoid solutions grew into hexagonal shapes and maintained that shape until crystals grew outside of the microscope sight. These findings suggested that all glycopolypeptoids could interact with the ice crystal surface through adsorption–inhibition, thereby regulating ice crystal morphology.

### 3.3. Effect of Glycopolypeptoids on Ice Crystal Growth Rate

**Figure 5:**
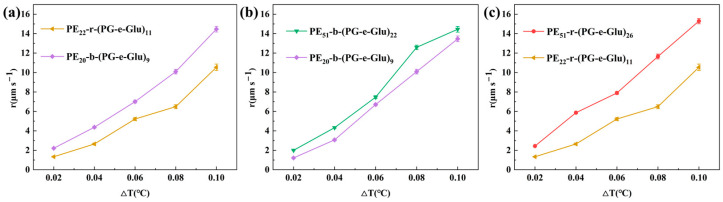
The effects of glycopolypeptoid structures on ice crystal growth rate under different supercooling temperatures (at a concentration of 2 mg mL^−1^): (a) samples PE_22_-r-(PG-e-Glu)_11_ and PE_20_-b-(PG-e-Glu)_9_; (**b**) samples PE_51_-b-(PG-e-Glu)_22_ and PE_20_-b-(PG-e-Glu)_9_; (**c**) samples PE_51_-r-(PG-e-Glu)_26_ and PE_22_-r-(PG-e-Glu)_11_.

**Figure 6:**
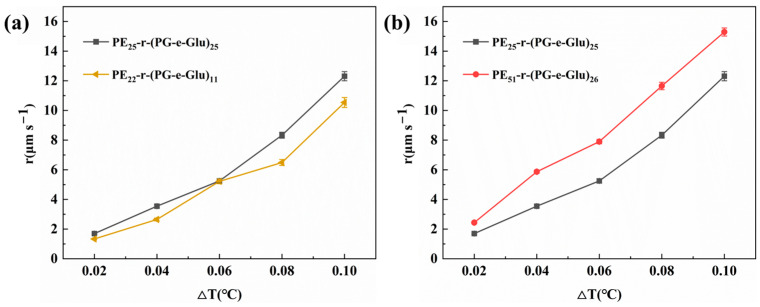
The effects of glycopolypeptoid hydrophobic and hydrophilic units on the ice crystal growth rate under different supercooling temperatures at a concentration of 2 mg mL^−1^: (**a**) samples PE_25_-r-(PG-e-Glu)_25_ and PE_22_-r-(PG-e-Glu)_11_; (**b**) samples PE_25_-r-(PG-e-Glu)_25_ and PE_51_-r-(PG-e-Glu)_26_.

Glycopolypeptoids with varying main-chain structures (block and random hydrophobic/hydrophilic unit sequences with different hydrophobic/hydrophilic unit compositions), different molecular weights, and different sugar side chains were prepared to study their effects on ice crystal growth at varying ΔTs (Figure 5 and Figure 6).

As shown in Figure 5a, the effects of the hydrophobic/hydrophilic unit sequence of glycopolypeptoids on the ice crystal growth rate were compared. Samples PE_22_-r-(PG-e-Glu)_11_ and PE_20_-b-(PG-e-Glu)_9_ possessed the same side sugar group and almost the same DPs for both hydrophobic and hydrophilic units but had a different sequence; PE_22_-r-(PG-e-Glu)_11_ was the random sequence and PE_20_-b-(PG-e-Glu)_9_ was the block sequence. Clearly, the ice grew slower in the PE_22_-r-(PG-e-Glu)_11_ solution than that in the PE_20_-b-(PG-e-Glu)_9_ solution, which suggested that a random sequence could better inhibit ice crystal growth. The likely reason was that the random sequence of hydrophobic/hydrophilic units matched the ice crystal lattice better, thus could be better absorbed onto the ice surface, resulting in slower ice growth.

In addition, the inhibition effect of glycopolypeptoids on their ice crystal growth rate at different DPs was further investigated. Samples PE_51_-b-(PG-e-Glu)_22_ and PE_20_-b-(PG-e-Glu)_9_ (Figure 5b) had the same side sugar group and block main-chain structure and almost the same hydrophobic/hydrophilic unit composition but the latter had a smaller DP. The ice in the PE_20_-b-(PG-e-Glu)_9_ solution grew slightly slower than that in the PE_51_-b-(PG-e-Glu)_22_ solution. Randomly sequenced glycopolypeptoids (PE_51_-r-(PG-e-Glu)_26_ and PE_22_-r-(PG-e-Glu)_11_) (Figure 5c) were also compared, and the ice in the PE_22_-r-(PG-e-Glu)_11_ solution clearly grew more slowly. It could be concluded that glycopolypeptoids with smaller DPs could slow down ice crystal growth more, regardless of block or random sequences.

Randomly sequenced glycopolypeptoids were employed to study the effect of hydrophobic and hydrophilic DPs on the ice crystal growth rate. PE_25_-r-(PG-e-Glu)_25_ and PE_22_-r-(PG-e-Glu)_11_ had comparative amounts of hydrophobic units but PE_22_-r-(PG-e-Glu)_11_ had fewer hydrophilic units. Figure 6a shows that there was no significant pattern of ice growth rate in these two glycopolypeptoid solutions, suggesting that the DP of the hydrophilic units had little effect on the ice crystal growth inhibition ability. PE_25_-r-(PG-e-Glu)_25_ and PE_51_-r-(PG-e-Glu)_26_ had comparative amounts of hydrophilic units but a different amount of hydrophobic units. Clearly, PE_25_-r-(PG-e-Glu)_25_, with fewer hydrophobic units, could better inhibit ice crystal growth (Figure 6b). It was suggested that the amount of hydrophobic units had more effect on the ice growth rate and that fewer hydrophobic units could reduce the rate more, thus being preferential because of better antifreeze properties.

**Figure 7:**
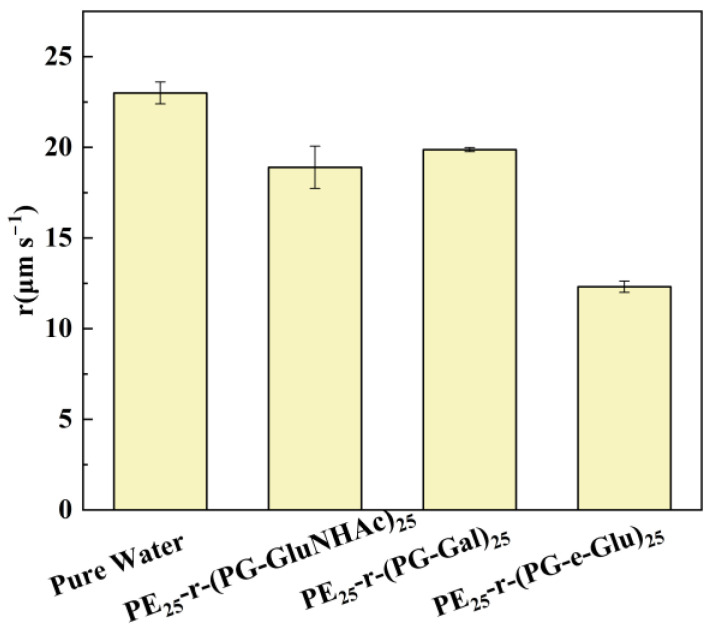
The effects of different kinds of glycopolypeptoids on the ice crystal growth rate (ΔT = 0.1 °C; 2 mg mL^−1^).

Furthermore, different sugars were connected onto polypeptoid side chains to study the effect of sugar side chains as the hydrophilic units on the ice crystal growth rate. The ice crystal growth rates of PE_25_-r-(PG-GluNHAc)_25_, PE_25_-r-(PG-Gal)_25_, and PE_25_-r-(PG-e-Glu)_25_ at 0.1 °C ΔT were 18 µm s^−1^, 19 µm s^−1^, and 13 µm s^−1^, respectively. Compared with pure water (23 µm s^−1^), all three glycopolypeptoids with varying sugars could inhibit the growth of ice crystals. Among the three glycopolypeptoids, PE_25_-r-(PG-e-Glu)_25_ had the best ability to slow down ice crystal growth (Figure 7).

### 3.4. Effect of Glycopolypeptoids on Ice Recrystallization Inhibition (IRI) Activity

**Figure 8:**
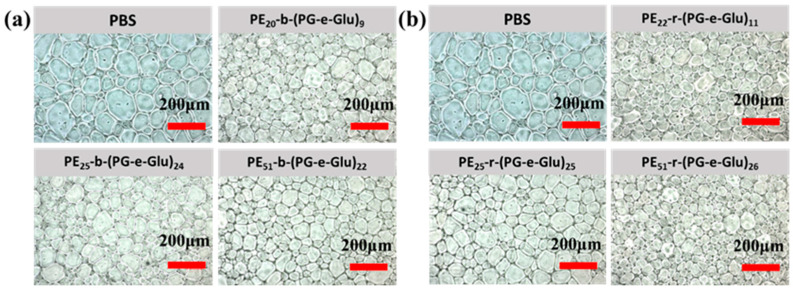
The electron microscope photos of ice crystals (annealed at −8 °C for 30 min; PBS solution is experimental control): (**a**) the block glycopolypeptoid solution; (**b**) the random glycopolypeptoid solution.

The IRI activity of the glycopolypeptoids was quantitatively evaluated using a “splat” analysis. In this method, photographs of ice crystals were taken after 30 min of nucleation and growth at −8 °C. The results of IRI activity were demonstrated by MLGS percentages. Figure 8a and b show the ice crystal micrographs of the block glycopolypeptoid solution and the random glycopolypeptoid solution, respectively. It can clearly be seen that the ice crystal sizes of both the block and random glycopolypeptoid solutions were smaller than those of the PBS control, which suggested that glycopolypeptoids had the ability to inhibit ice recrystallization.

**Figure 9:**
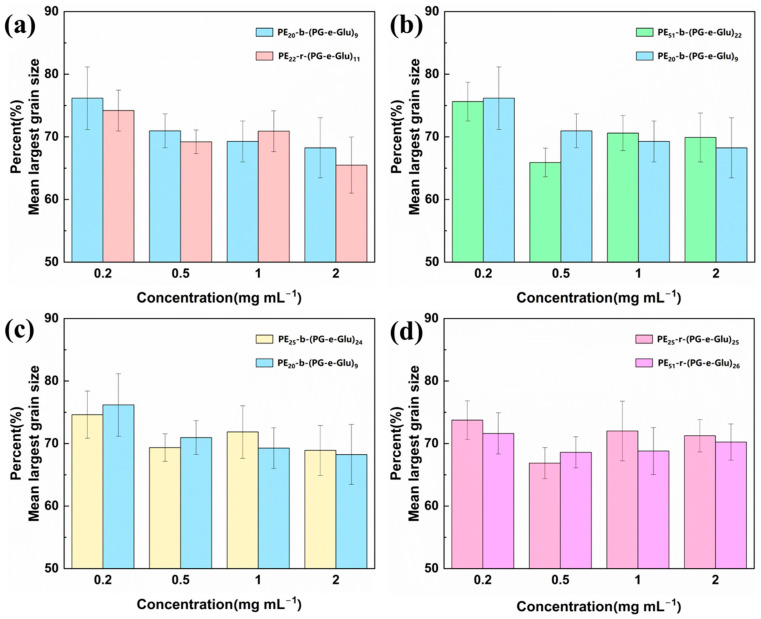
MLGS percentages of ice crystals of glycopolypeptoids with different structures in PBS solutions:(**a**) PE_20_-b-(PG-e-Glu)_9_ and PE_22_-b-(PG-e-Glu)_11_, (**b**) PE_51_-b-(PG-e-Glu)_22_ and PE_20_-b-(PG-e-Glu)_9_, (**c**) PE_25_-b-(PG-e-Glu)_24_ and PE_20_-b-(PG-e-Glu)_9_, and (**d**) PE_25_-r-(PG-e-Glu)_25_ and PE_51_-b-(PG-e-Glu)_26_.

The MLGS percentages of samples with different concentrations were measured to investigate the effects of glycopolypeptoid main-chain structures and degrees of polymerization on IRI activity. Generally, when the MLGS or MLGS percentage is smaller, IRI activity is better. As shown in Figure 9, overall, the MLGS percentages decreased with an increase in the glycopolypeptoid concentration. PE_22_-r-(PG-e-Glu)_11_ at a concentration of 2 mg mL^−1^ had the best IRI activity and the MLGS percentage was reduced to 64.8 ± 4.4%. These results indicated that the IRI activity of glycopolypeptoids was positively correlated with the concentration. The effects of the unit sequence, degree of polymerization, and unit composition on IRI activity (Figure 9) were also studied. No obvious differences or trends were observed, suggesting all glycopolypeptoids had comparable IRI activity.

**Figure 10:**
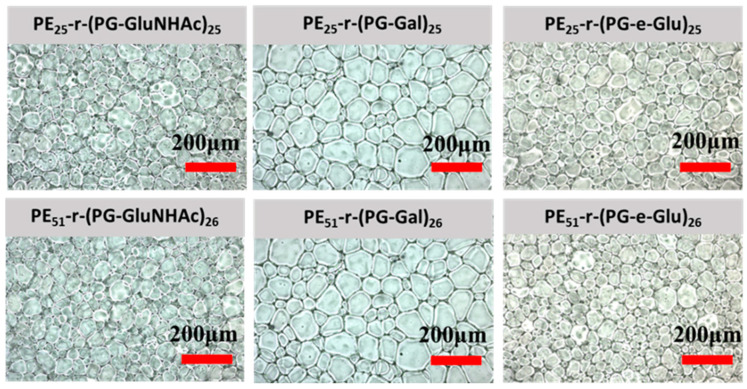
The microscope photos of different glycopolypeptoids (annealed at −8 °C for 30 min; PBS solution is experimental control).

The effects of different kinds of sugar side chains and hydrophobic units amounting to the IRI activity of the glycopolypeptoids were also studied. As shown in Figure 10, the sizes of the ice crystals in the presence of all glycopolypeptoids were much smaller than those of the PBS control, indicating that all the glycopolypeptoids possessed the ability to inhibit ice recrystallization.

**Figure 11:**
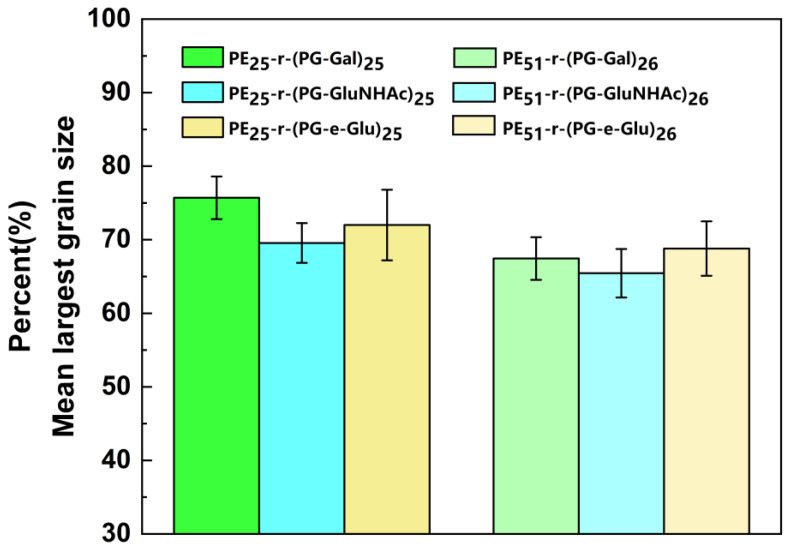
MLGS percentages of ice crystals of glycopolypeptoids with different sugar groups and different amount of hydrophobic units in PBS solutions.

Figure 11 shows the MLGS percentages of six different glycopolypeptoids to evaluate their IRI activity. The difference between the hydrophilic units, i.e., the sugar structures, did not have an obvious effect on the IRI activity of glycopolypeptoids. In the nanoliter osmometry experiment, fewer hydrophobic units were preferred to slow down ice growth. In Figure 11, however, glycopolypeptoids with different amount of hydrophobic units had similar IRI activity, regardless of the hydrophilic unit structures (sugar types).

### 3.5. Cytotoxicity Tests of Glycopolypeptoids

**Figure 12:**
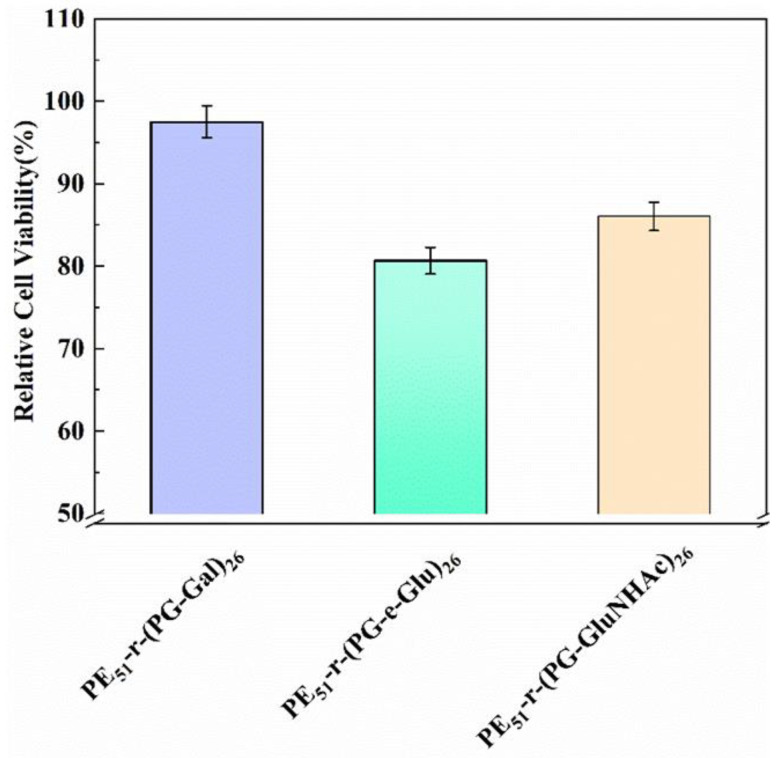
Percentage of relative cell viability between glycopolypeptoid treatments and control group.

In order to verify the safety of the synthesized glycopolypeptoids, the three above-mentioned random glycopolypeptoids were selected for cytotoxicity experiments. As shown in Figure 12, the relative cell viabilities of PE_51_-r-(PG-Gal)_26_, PE_51_-r-(PG-e-Glu)_26_, and PE_51_-r-(PG-e-GluNHAc)_26_ were 97.5%, 80.7%, and 86.1%, respectively. These glycopolypeptoids could be considered to be non-cytotoxic because all the relative cell viabilities were above 75% [45], confirming their potential utilization as biomedical materials.

## 4. Conclusions

In this study, glycopolypeptoids were synthesized and investigated for their antifreeze properties for the first time. Through a controlled polymerization process followed by click chemistry, glycopolypeptoids with tunable structures were successfully designed and prepared. Their antifreeze properties were systematically evaluated using nanoliter osmometry and ice recrystallization inhibition assays, demonstrating that these synthetic polymers could effectively modulate ice formation and growth. The prepared glycopolypeptoids were able to tune the ice crystal morphology, slow down ice growth, and inhibit ice recrystallization, indicating their potential as novel cryoprotectants. The study also revealed a strong correlation between the molecular structure and antifreeze performance. A random sequence of hydrophobic/hydrophilic units, a smaller DP, and fewer hydrophobic units are preferential to achieve better antifreeze activity. These findings provide valuable insights into the design of biomimetic antifreeze materials, and pave the way for their application in cryopreservation across biomedical, pharmaceutical, and industrial fields.

## Figures and Tables

**Table 1:** The prepared polypeptoids copolymers.

Polymer	[M_1_]_0_:[M_2_]_0_:[I]_0_ ^a^	M_n_ (NMR) (kg mol^−1^) ^b^	M_n_ (GPC) (kg mol^−1^) ^c^	PDI ^c^	Yield
PNEG_20_-b-PNPG_9_	20:10:01	2.6	35.9	1.06	80%
PNEG_25_-b-PNPG_24_	25:25:01	4.4	48.1	1.09	85%
PNEG_51_-b-PNPG_22_	50:25:01	6.5	55	1.1	81%
PNEG_22_-r-PNPG_11_	20:10:01	3	35.5	1.05	86%
PNEG_25_-r-PNPG_25_	25:25:01	4.5	44.8	1.05	80%
PNEG_51_-r-PNPG_26_	50:25:01	6.9	55.8	1.04	88%

^a^ Feed molar ratio of NCA/initiator; ^b^ calculated from ^1^H NMR; ^c^ determined from GPC.

## Data Availability

The original contributions presented in this study are included in the article/Supplementary Material. Further inquiries can be directed to the corresponding authors.

## Supplementary Materials

The following supporting information can be downloaded at: https://www.mdpi.com/article/10.3390/polym17121600/s1, Figure S1: ^1^H NMR spectrum of Ethyl-NCA (400 MHz, CDCl_3_); Figure S2: ^1^H NMR spectrum of Propynyl-NCA (400 MHz, CDCl_3_); Figure S3: FT-IR spectra of the polypeptoids (red), Ethyl-NCA (blue) and Propynyl-NCA (yellow); Figure S4: ^1^H NMR spectrum of PNEG_51_ (400 MHz, CDCl_3_); Figure S5: ^1^H NMR spectrum of PNEG_51_-b-PNPG_22_ (400 MHz, CDCl_3_); Figure S6: GPC calibration curve with polystyrene standards; Figure S7: ^1^H NMR spectrum of glycopolypeptoids PE_51_-b-(PG-e-Glu)_22_ (400 MHz, D_2_O); Table S1: The yields of all glycopolypeptoids.
