# Supplementary material for: Glycopolypeptoids as Novel Biomimetic Antifreeze Agents: Structural Design, Synthesis, and Antifreeze Properties
Source: Polymers (Basel). 2025 Jun 8;17(12):1600. doi: 10.3390/polym17121600 (PMC12197131; doi:10.3390/polym17121600)
Supplement: Supplementary file 1 [file polymers-17-01600-s001.zip › polymers-3636187-supplementary.pdf]

Supporting Information

Glycopolypeptoids as Novel Biomimetic Antifreeze Agents: Structural Design, Synthesis, and Antifreeze Properties

Liugen Xu †, Junwei Pi †, Lei Feng, Junhao Wen, Minghai Zhao, Jianwei Lu, Amjad Ali, Li Guo*

School of Materials Science and Engineering, Jiangsu University, Zhenjiang, 212013, China

† Authors contribute equally to this work.

Correspondence to: Li Guo (Email: liguo@ujs.edu.cn)

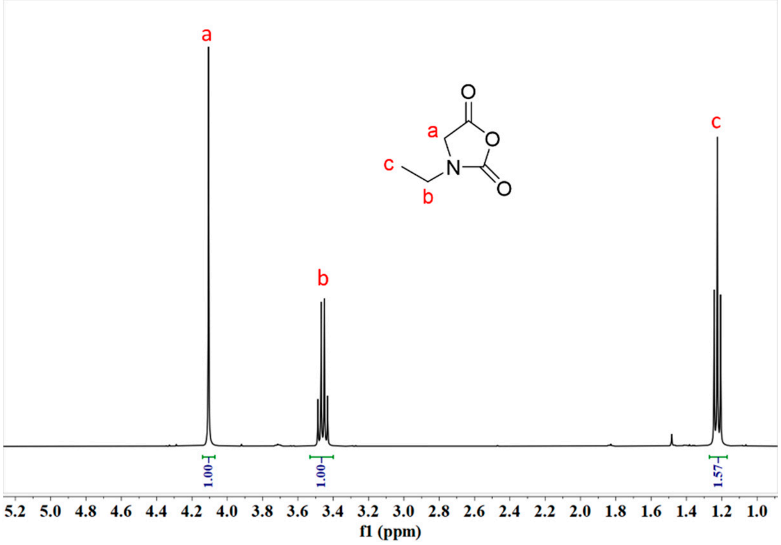

Figure S1. ^{1}H NMR spectrum of Ethyl-NCA. (400 MHz, $CDCl_3$)

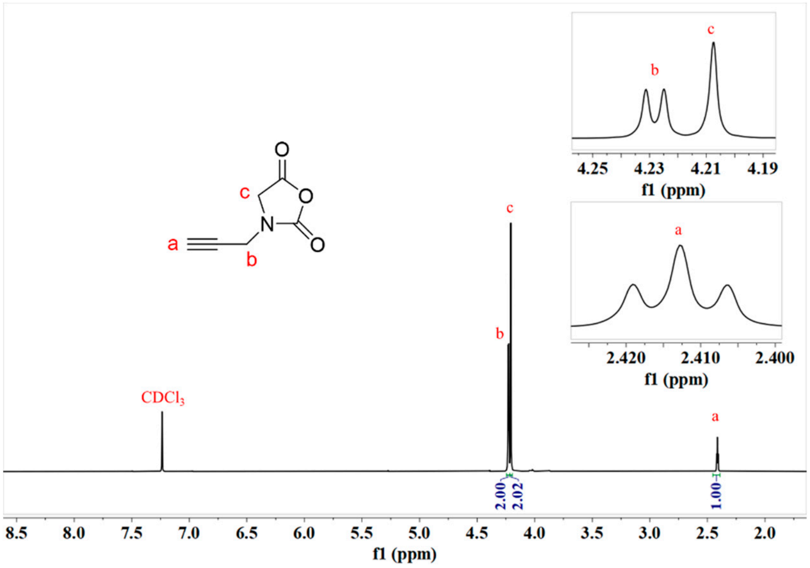

Figure S2. ^{1}H NMR spectrum of Propynyl-NCA. (400 MHz, $CDCl_3$)

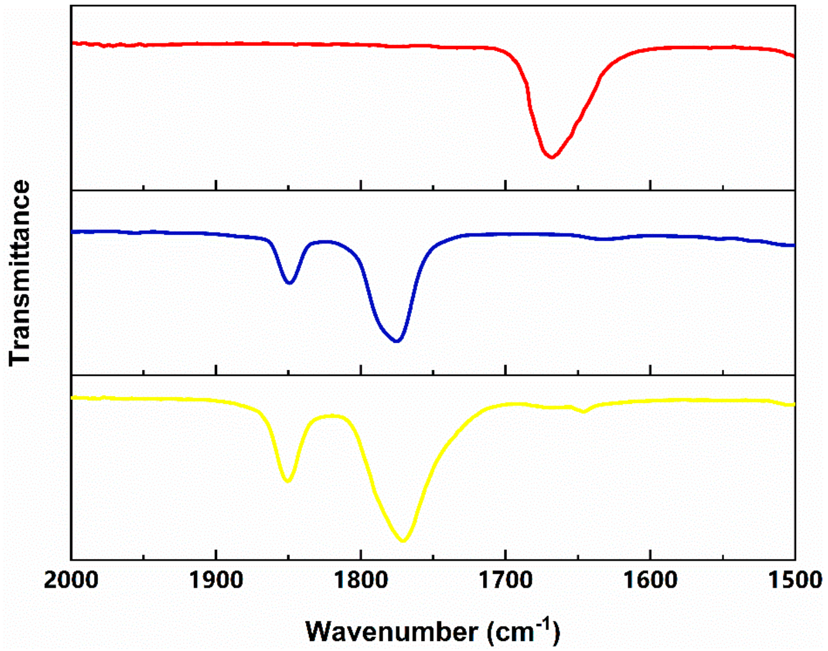

Figure S3. FT-IR spectra of the polypeptoids (red), Ethyl-NCA (blue) and Propynyl-NCA (yellow).

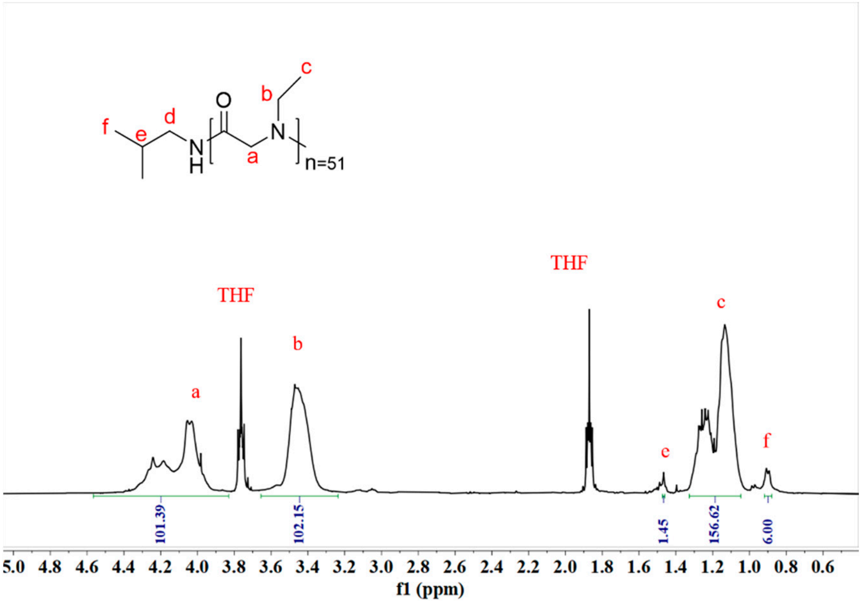

Figure S4. ^{1}H NMR spectrum of $PNEG_{51}$. (400 MHz, $CDCl_3$)

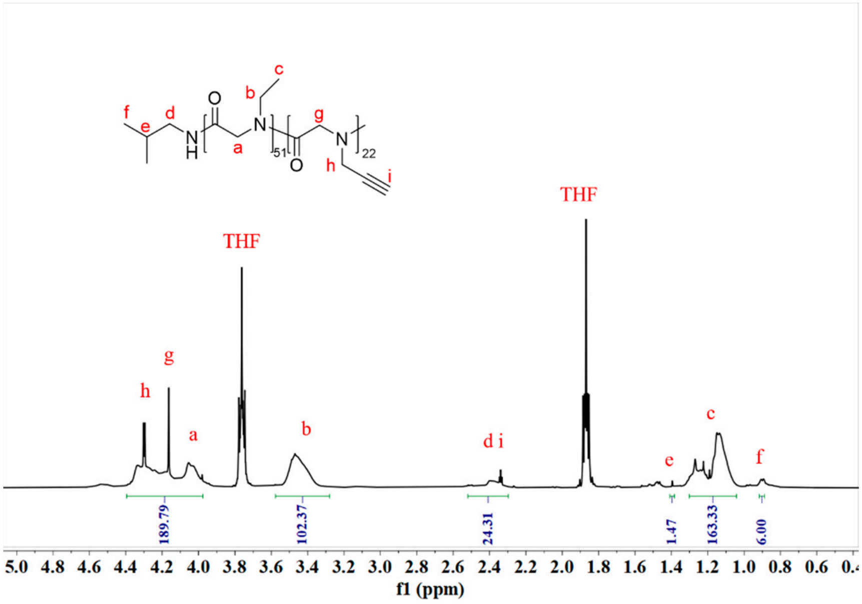

Figure S5. ^{1}H NMR spectrum of $PNEG_{51}$-b-$PNPG_{22}$. (400 MHz, $CDCl_3$)

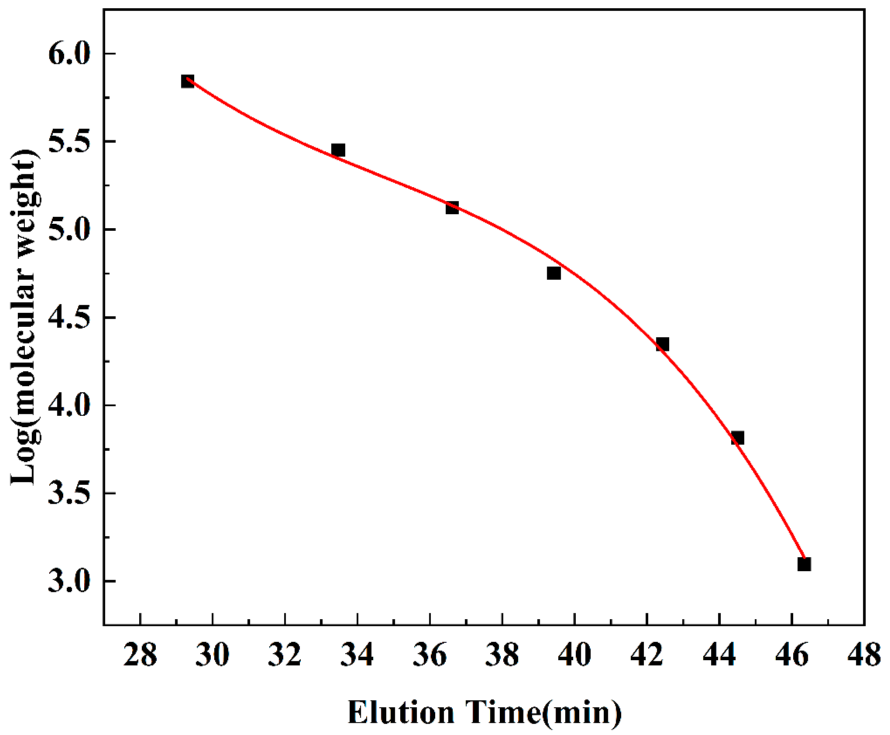

Figure S6. GPC calibration curve with polystyrene standards.

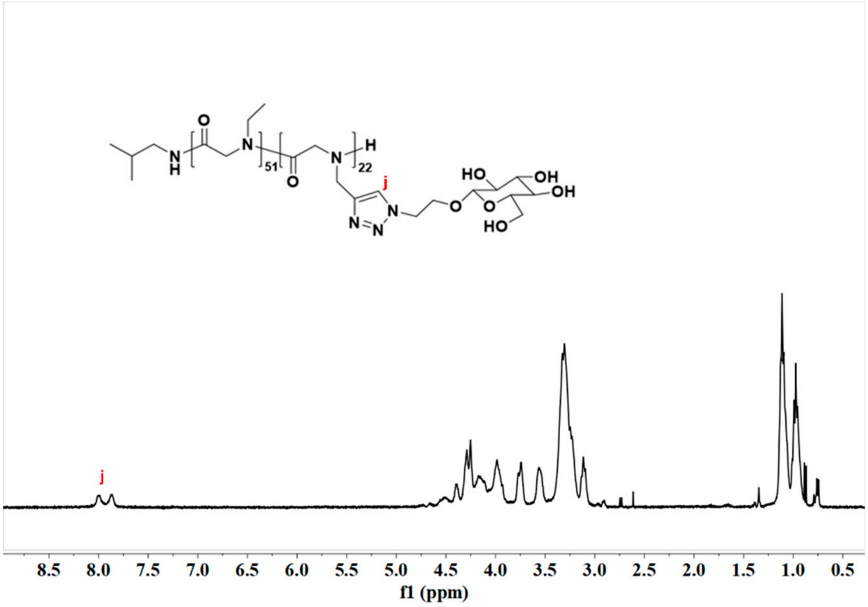

Figure S7. ^{1}H NMR spectrum of glycopolypeptoids PE_{51}-b-(PG-e-Glu)$_{22}$. (400 MHz, D_2O).

Table S1. The yields of all glycopolypeptoids.

Polymer	Yield
PE_{25}-r-$(PG\text{-}Gal)_{25}$	69%
PE_{51}-r-$(PG\text{-}Gal)_{26}$	64%
PE_{25}-r-$(PG\text{-}GluNHAc)_{25}$	65%
PE_{51}-r-$(PG\text{-}GluNHAc)_{26}$	62%
PE_{25}-r-$(PG\text{-}e\text{-}Glu)_{25}$	67%
PE_{51}-r-$(PG\text{- }e\text{-}Glu)_{26}$	70%
